# The relationship between work practice environment and work engagement among nurses: The multiple mediation of basic psychological needs and organizational commitment a cross-sectional survey

**DOI:** 10.3389/fpubh.2023.1123580

**Published:** 2023-03-07

**Authors:** Yun-xia Ni, Ya Wen, Ying Xu, Li He, Gui-ying You

**Affiliations:** West China School of Nursing, Sichuan University/Department of Cardiology, West China Hospital, Sichuan University, Chengdu, China

**Keywords:** basic psychological needs, mediation, nurses, organizational commitment, work engagement, work practice environment

## Abstract

**Background:**

Previous researchers have demonstrated that the work practice environment influences nurses' engagement; however, few studies have explored the mechanisms that explain the links between them.

**Objectives:**

To examine whether basic psychological needs and organizational commitment mediate the relationship between the work practice environment and work engagement.

**Methods:**

A cross-sectional survey was conducted with a sample of 893 nurses from 14 cities in Sichuan Province of China between November 2021 and December 2021. Data were collected online using the Chinese version of the Practice Environment Scale of the Nursing Work Index, Basic Needs Satisfaction in General Scale, Organizational Commitment Scale, and Utrecht Work Engagement Scale. The Pearson correlation analysis and multiple mediation model were used to analyze the data.

**Results:**

The Pearson correlation analysis showed that work practice environment, basic psychological needs, and organizational commitment were positively associated with work engagement. The positive relationship between work practice environment and work engagement was mediated by basic psychological needs and organizational commitment [*B* = 0.505, *SE* = 0.032, 95% *CI* (0.442, 0.566)].

**Conclusions:**

The study substantially contributes to the existing knowledge by revealing the mechanisms of fostering work engagement among nurses and explaining why the work practice environment influences work engagement.

## Introduction

Nurses make up a significant portion of healthcare professionals worldwide (1), including in China. Accordingly, the quality of care provided to individuals within healthcare organizations may largely rely on nurse performance (1). Work engagement has emerged as a crucial topic for nurses' performance (2). Work engagement is defined as “…a positive, fulfilling work-related state of mind, and characterized by vigor, dedication and absorption” (3). A growing body of research (2, 4, 5) has reported that work engagement improves various organizational-related outcomes and personal-related outcomes, including quality of care, patient satisfaction, nurse health (e.g., subjective wellbeing, mental health, physical health), and turnover intention. Nurses with a higher level of work engagement are more likely to be enthusiastic about their work and immersed in their task, which in turn contributes to desirable performance (5). Instead, nurses with a lower level of work engagement tended to report negative performance, such as absenteeism, missed nursing care, or even intention to leave (4). Accordingly, how to improve nurses' work engagement has captured wide attention worldwide.

To develop effective strategies that foster work engagement, identifying potential predictors of work engagement is imperative. Previous studies have shown that the antecedents of work engagement are diverse (6, 7). For example, Zahari et al. (7) summarized 48 articles related to work engagement and found that the predictors of work engagement could be divided into five categories, namely, organizational and team factors, perceived leadership, organizational intervention, job-related experience, and individual factors. Another review conducted by Kato et al. (6) indicated that job resources (e.g., organizational factors, interpersonal relationship factors, and task factors) and job demands (e.g., quantitative workload) are the main factors that influence work engagement. Among these antecedents of work engagement, many are obviously related to or even components of the work practice environment, such as leadership, support from organizations, and interpersonal relationships. The work practice environment is a multidimensional concept that refers to the organizational characteristics of a work setting that facilitate or hinder nursing practice (8), including nurse participation in hospital affairs; nursing foundations for quality of care; nurse manager ability, leadership and support of nurses; staffing and resource adequacy; and collegial nurse–physician relationships. There is an extensive body of research showing that the work practice environment is associated with work engagement across different cultures (9, 10). For example, Li et al. (10) found that a favorable work practice environment (e.g., nurse–physician relations, nurse manager ability and leadership) was positively linked to higher work engagement. Conversely, a negative work practice environment, for instance, toxic leadership (e.g., abusive, intemperate, narcissistic, or humiliating), was strongly associated with poor outcomes such as lower job involvement and decreased quality of care (11). In particular, under the unprecedented COVID-19 pandemic, nurses face a challenging work practice environment, such as a nursing shortage, long working days, and insufficient protective equipment supply (12). How this challenging work practice environment affects nurses' work engagement under COVID-19 working conditions remains unclear. Moreover, the underlying mechanism between the work practice environment and work engagement has been explored far less.

Previous studies have found that basic psychological needs act as an important predictor of work engagement (13, 14). Ni et al. (13) reported a positive link between basic psychological need satisfaction and work engagement among nurses. Ryan and Deci (15) suggested that people have three kinds of intrinsic basic psychological needs: autonomy (the desire to experience freedom and make choices in one's actions), competence (the desire to feel capable of achieving desired outcomes), and relatedness (the feeling of belongingness and connectedness with others). Once these psychological needs are satisfied, individuals could be motivated to devote more time and effort to their task and feel full of physical energy, which is known as work engagement (15). Due to the work context and medical personnel's professionalism, nurses' satisfaction with three basic psychological needs may be derived from their perceptions of the work environment, such as a sense of competence at work and a sense of belonging when leaders care about them (14). Several empirical studies have demonstrated that a supportive work practice environment can satisfy individuals' three innate psychological needs (14, 16). For example, Malhotra et al. (16) showed that organizational justice was a significant predictor of individuals' psychological needs satisfaction. Likewise, a cross-sectional study conducted in Indonesian employees demonstrated that engaging leadership, a component of the work practice environment, had a positive impact on basic psychological need satisfaction (14). Given that the link between the work practice environment and basic psychological need satisfaction, as well as the association between basic psychological need satisfaction and work engagement, have been reported by previous empirical studies, we speculated that the fulfillment of basic psychological needs may act as a mediator in the relationship between the work practice environment and work engagement.

Social Exchange Theory (SET) may serve as a theoretical basis that helps to understand the relationship among the work practice environment, basic psychological need satisfaction, and work engagement. SET is one of the prominent underpinning theories that has been frequently used in previous studies to explain individuals' work engagement (7). These studies selected SET based on the assumption that work engagement is affected by workplace interactions between organizations and employees (17). The basic premise of SET was that the employee-organizational relationship was formed based on subjective cost–benefit analysis (18). If employees experience trust, respect, and support from their organizations, a favorable employee-organizational relationship is created, and employees may choose to reciprocate these treatments with positive attitudes and good behavior in their work (18). As an important characteristic of organizations, the work practice environment plays a crucial role in creating employee-organizational relationships. When organizations provide a supportive environment (e.g., learning opportunities, cooperative interactions, and emotional support), a positive employee-organizational relationship is established, and employees' basic psychological needs are more likely to be fulfilled, which in turn promotes their engagement in work (7).

Organizational commitment is a well-studied concept reflecting the employee-organizational relationship, which refers to one's feelings of attachment toward a particular organization (19). Past studies have shown that a favorable work practice environment may lead to a higher organizational commitment (20). Seren Intepeler et al. (20) confirmed that positive work environments, such as leadership support, positive team relationships, and nurses' participation in decision-making, had a positive impact on individuals' commitment to an organization. According to SET, when work practice environments allow employees to experience a high level of organizational commitment, positive and mutual reciprocity is created, which in turn makes individuals feel obligated to repay their organizations, such as investing more energy in their work, and become more engaged. On the other hand, a positive link between organizational commitment and work engagement has been reported by prior studies (21, 22). A study by Gupta et al. (22), with a sample of 750 nurses in India, indicated that a high level of commitment could improve work engagement. Kim et al. (23) summarized the research related to work engagement and found that commitment could be the precursor to engagement. Despite the lack of research confirming the trilateral relationship among the work practice environment, organizational commitment, and work engagement, drawing on SET and previous studies, it is reasonable to infer that organizational commitment may serve as a mechanism linking the work practice environment and work engagement.

**Figure 1:**
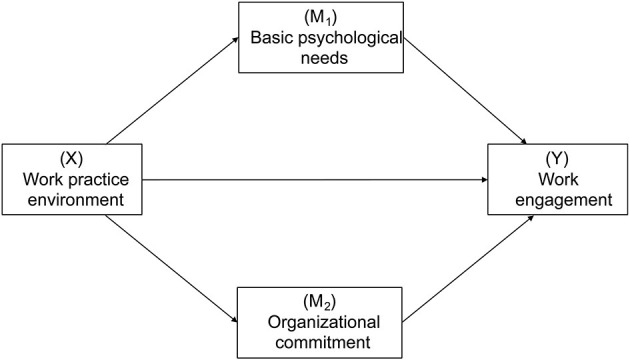
The hypothesized model.

To date, it is still unclear why the work practice environment influences work engagement in the nursing context. It is of great significance to reveal the relationships among the work practice environment, basic psychological needs, organizational commitment, and work engagement since these relationships could be helpful to guide strategy development to improve nurses' engagement at work and ultimately improve nurse performance and quality of care. According to the theoretical analyses and empirical research, the hypothesized model was proposed (see Figure 1) to examine the multiple mediation effects of basic psychological needs and organizational commitment on the relationship between the work practice environment and work engagement among nurses. Consequently, the following hypotheses were proposed:

Hypothesis 1: Work practice environment is positively related to nurses' work engagement.

Hypothesis 2: Basic psychological needs mediate the relationship between the work practice environment and work engagement.

Hypothesis 3: Organizational commitment mediates the relationship between the work practice environment and work engagement.

## Materials and methods

### Design, participants, and settings

A descriptive, cross-sectional design was applied in this study.

The following inclusion criteria were used: (a) age ≥ 18 years old; (b) registered nurse; (c) working in the current hospital for at least 1 year; and (d) voluntary participation in the study. The exclusion criteria were (a) practice nurses and trainee nurses without practice qualifications and (b) nurses who were absent from work during the survey period (e.g., studying abroad, on holiday, sick or maternity leave, or asking for leave).

Following Fritz's recommendation (24) and our data analysis method (i.e., bootstrapping method), the sample size in our study should be more than 462. Considering a possible non-response rate of 20%, the minimum sample size should be 578. We distributed 955 questionnaires, of which 893 were returned, thus yielding a response rate of 93.5%.

This online survey was conducted in Sichuan Province located in the southwest region of China. Stratified convenience sampling was used to recruit participants. Stratification was based on geographical region and economic development. Sichuan Province is divided into five areas: the central, southern, northeastern, western, and northwest regions. Each area included two to eight cities. A three-stage sampling method was used to recruit the participants in the study. First, a total of 14 cities were selected randomly from a total of 21 cities. Among those selected, there were five cities from the central region, three from the southern region, three from the northeastern region, two from the western region, and one from the northwest region. Second, the hospitals were selected conveniently based on the feasibility of the study. Third, nurses in each selected hospital were recruited conveniently.

### Data collection

The data collection was carried out from November to December 2021. To reduce mobility and maintain social distance during the COVID-19 pandemic, we collected data through an online survey. The electronic questionnaire was developed on the widely used online platform called Wenjuanxing, which could generate a website link to the electronic questionnaire. Before the survey, the nursing administrators of each hospital were contacted *via* phone to explain the study aims and procedures and inform the meaning of our study. After obtaining permission, we sent the website link to the questionnaire to nursing administrators *via* WeChat, which is the most popular social media platform in China. Questionnaires were distributed to nurses who met the inclusion criteria by nursing administrators *via* the WeChat group. At the beginning of the questionnaire, brief information about the survey purpose, significance, and filling method of the questionnaire was given to the participants, and they were required to sign informed consent forms (click a “box” to agree to participate in this survey) before enrollment. Participants completed questionnaires by themselves and submitted them through WeChat anonymously; thus, nursing administrators could not see the completed questionnaire. All data could not be accessed by others except the researchers. The researchers checked each collected questionnaire and excluded the questionnaires in which all the answers were the same.

### Variables and measures

The Chinese version (25) of the Practice Environment Scale of the Nursing Work Index (PES-NWI) (8) was used to measure nurses' perceptions of their work practice environment. The original PES-NWI consists of 31 items, and the Chinese version consists of 28 items that measure five components of the work practice environment: nurse participation in hospital affairs (eight items); nursing foundations for quality of care (nine items); nurse manager ability, leadership and support of nurses (four items); staffing and resource adequacy (four items); and collegial nurse–physician relationships (three items). Each item was scored on a 4-point Likert scale ranging from one (strongly disagree) to four (strongly agree), and a higher score represented a more supportive work practice environment that nurses perceived. The average score of the whole scale was calculated by adding the scores obtained in all items and then dividing by the total number of items. The test-retest reliability of the Chinese version of the PES-NWI was 0.84, and the content validity was 0.94 (25). The Cronbach's alpha for this scale was 0.97 in our study.

The Chinese version of the Basic Needs Satisfaction in General Scale (BNSG-S) was used to evaluate the extent to which a person satisfied with basic psychological needs (26). The original BNSG-S was modified by Liu to accommodate the working context in China. The BNSG-S consists of 21 items that measure three dimensions of needs: autonomy (seven items), competence (six items), and relatedness (eight items). Each item was scored on a 7-point Likert scale ranging from one (strongly disagree) to seven (strongly agree), and higher scores indicated higher satisfaction of basic psychological needs. The average score of the whole scale was calculated by adding the scores obtained in all items and then dividing by the total number of items. The BNSG-S showed good validity and reliability among nurses (27). The Cronbach's alpha for the BNSG-S was 0.90 in our study.

The Chinese version of the Organizational Commitment Scale was used to assess the extent to which a person committed to their organizations (28). This 18-item scale measures three dimensions of organizational commitment: affective commitment (feeling of identification with and emotional attachment to the organization) (six items), normative commitment (feelings of cost related to leaving the organization) (six items), and continuance commitment (feeling of obligation to the organization) (six items) (29). Each item was scored on a 5-point Likert scale ranging from one (strongly disagree) to five (strongly agree), and higher scores represented high levels of commitment to the organization by the nurses. The average score of the whole scale was calculated by adding the scores obtained in all items and then dividing by the total number of items. This scale has been widely used among Chinese nurses and has shown good validity and reliability (30). In this study, the Cronbach's alpha of the total scale was 0.92, and for each dimension, it was between 0.78 and 0.96.

The Chinese version (31) of the 9-item Utrecht Work Engagement Scale (UWES) was used to measure work engagement (32). This scale measures three components of work engagement: vigor (three items), dedication (three items), and absorption (three items). Each item was scored on a 7-point scale ranging from 0 (never) to 6 (always), and higher scores indicated greater engagement. The average score of the whole scale was calculated by adding the scores obtained in all items and then dividing by the total number of items. The Chinese version of the UWES showed good validity and reliability among nurses (10). The Cronbach's alpha for work engagement was 0.95 in our study.

A self-deigned questionnaire was used to assess participants' demographic characteristics, such as age, gender, education, and years of nursing experience.

### Data analysis

As the data were self-reported, common method variance bias was examined based on Podsakoff et al. (33) recommendation. Harman's single factor test was first performed, followed by the comparison of the model fit indices between the hypothesized model (four-factor model) and the added common-method factor (one-factor model). Nine factors had eigenvalues higher than one, and the first factor explained 31.7% of the total variance. The model fit indices of the hypothesized model were better than those of the added common-method factor (Δχ^2^ = 4431.217, *P* < 0.001). These results indicated that common method bias was not a major problem in our study. Moreover, the tolerance and variance inflation factor (VIF) were calculated to test the collinearity between variables. The results showed that the tolerance value was higher than 0.2, and the VIF value was smaller than 10, indicating that there was no serious collinearity problem (34).

Descriptive statistics (i.e., frequencies, percentages, or means and standard deviations) were used to describe the demographics of participants and the four key study variables (work practice environment, basic psychological needs, organizational commitment, and work engagement) according to the data type. The Shapiro–Wilk test and Q-Q plot were used to assess normality. The association between the key study variables was examined using Pearson correlation analysis. Research hypotheses were simultaneously tested in a single model by using the PROCESS (version 4.0) macro for SPSS provided by Hayes (35), which was based on ordinary least-squares regression. Following Hayes' recommendations (35), the mediation effects were calculated through a bootstrapping method (with 5,000 resamples), which was considered the most powerful test of mediation compared with the Baron and Kenny method (35). The mediation effect is significant if the values of the lower and upper limits of the 95% confidence interval (CI) do not include “zero” (35). The PROCESS macro was performed using one independent variable (work practice environment), two mediators (basic psychological needs and organizational commitment), and one dependent variable (work engagement). Both the direct and total effects were examined based on Mathieu and Taylor's suggestions (36). The demographic characteristics (i.e., gender, age, and years of nursing experience) of the nurses were included as covariates because these characteristics were reported as potential factors related to work engagement based on the literature (6). Data were analyzed using IBM SPSS statistics version 26.0 for Windows (IBM Corp., Armonk, NY, USA). The significance level of α was 0.05.

## Results

### Demographics of participants

The demographic characteristics of the participants are shown in Table 1. A total of 893 nurses participated in this study. The average age of the participants was 32.3 years (SD = 6.8), and the average number of years in nursing practice was 10.7 years (SD = 7.3). A majority of the participants (72.1%) were married, junior nurses (64.5%), and had a bachelor's degree (76.0%).

**Table 1:** Demographics of the participants (*N* = 893).

**Variable**	**Mean (SD)**	**Frequency (%)**
**Gender**		
Male		13 (1.5)
Female		880 (98.5)
**Age (years)**	32.3 (6.8)	
≤ 30		411 (46.0)
31–40		387 (43.3)
≥41		95 (10.6)
**Education**		
Secondary diploma		7 (0.8)
Advanced diploma		193 (21.6)
Bachelor's degree		679 (76.0)
Master's degree or higher		14 (1.6)
**Marital status**		
Single		221 (24.7)
Married		644 (72.1)
Divorced		28 (3.1)
**Years of nursing experience**	10.7 (7.3)	
≤ 5		225 (25.2)
6–10		335 (37.5)
11–15		158 (17.7)
>15		175 (19.6)
**Professional title**		
Junior		576 (64.5)
Intermediate		273 (30.6)
Senior		44 (4.9)

SD, standard deviation.

### Correlation analyses

The means, standard deviations, and correlations between the four key variables and their dimensions are shown in Table 2. The work practice environment was positively related to basic psychological needs (*r* = 0.66, *P* < 0.01), organizational commitment (*r* = 0.70, *P* < 0.01), and work engagement (*r* = 0.57, *P* < 0.01). Work engagement was positively correlated with basic psychological needs (*r* = 0.60, *P* < 0.01) and organizational commitment (*r* = 0.67, *P* < 0.01).

**Table 2:** Means, standard deviation, and correlations between the study variables (*N* = 893).

**Variables**	**Mean**	**SD**	**1**	**2**	**3**	**4**	**5**	**6**	**7**	**8**	**9**	**10**	**11**	**12**	**13**	**14**	**15**	**16**	**17**
1. WPE	3.27	0.49																	
2. WPE-NPHA	3.22	0.55	0.96																
3. WPE- NFQC	3.38	0.48	0.95	0.87															
4. WPE-NMALS	3.33	0.51	0.90	0.83	0.82														
5. WPE-SRA	3.05	0.64	0.86	0.78	0.74	0.73													
6. WPE-CNPR	3.32	0.56	0.88	0.79	0.82	0.79	0.70												
7. BPN	5.09	0.82	0.66	0.61	0.62	0.64	0.56	0.63											
8. BPN-AU	4.69	0.96	0.64	0.60	0.56	0.61	0.60	0.59	0.90										
9. BPN-CO	5.10	0.94	0.56	0.51	0.53	0.53	0.47	0.54	0.90	0.72									
10. BPN-RE	5.43	0.85	0.59	0.54	0.57	0.57	0.45	0.58	0.90	0.68	0.74								
11. OC	3.96	0.60	0.70	0.66	0.64	0.64	0.64	0.62	0.47	0.50	0.39	0.37							
12. OC-AC	4.23	0.69	0.79	0.74	0.77	0.74	0.67	0.73	0.63	0.61	0.54	0.54	0.87						
13. OC-NC	3.91	0.71	0.67	0.64	0.60	0.62	0.63	0.59	0.46	0.51	0.39	0.35	0.93	0.83					
14. OC-CC	3.73	0.71	0.32	0.30	0.29	0.29	0.33	0.27	0.11	0.15	0.08	0.05	0.76	0.39	0.54				
15. WE	4.30	1.37	0.57	0.52	0.50	0.51	0.56	0.54	0.60	0.60	0.55	0.48	0.57	0.64	0.57	0.25			
16. WE-VI	4.68	1.28	0.51	0.45	0.46	0.46	0.49	0.50	0.54	0.51	0.52	0.45	0.50	0.57	0.49	0.22	0.89		
17. WE-DE	3.90	1.76	0.47	0.45	0.40	0.42	0.48	0.45	0.51	0.52	0.46	0.39	0.50	0.55	0.51	0.22	0.89	0.67	
18. WE-AB	4.19	1.59	0.54	0.50	0.47	0.47	0.51	0.51	0.58	0.58	0.52	0.45	0.54	0.61	0.54	0.23	0.94	0.73	0.83

Correlations between variables were all significant (p < 0.01). SD, standard deviation; WPE, work practice environment; WPE–NPHA, work practice environment–nurse participation in hospital affairs; WPE–NFQC, work practice environment–nursing foundations for quality of care; WPE–NMALS, work practice environment–nurse manager ability, leadership and support of nurses; WPE–SRA, work practice environment–staffing and resource adequacy; WPE–CNPR, work practice environment–collegial nurse–physician relationships; BPN, basic psychological needs; BPN–AU, basic psychological needs–autonomy; BPN–CO, basic psychological needs–competence; BPN–RE, basic psychological needs–relatedness; OC, organizational commitment; OC–AC, organizational commitment–normative commitment; OC–NC, organizational commitment–normative commitment; OC–CC, organizational commitment–continuance commitment; WE, work engagement; WE–VI, work engagement–vigor; WE–DE, work engagement–dedication; WE–AB, work engagement–absorption.

### Test of mediation effects

**Figure 2:**
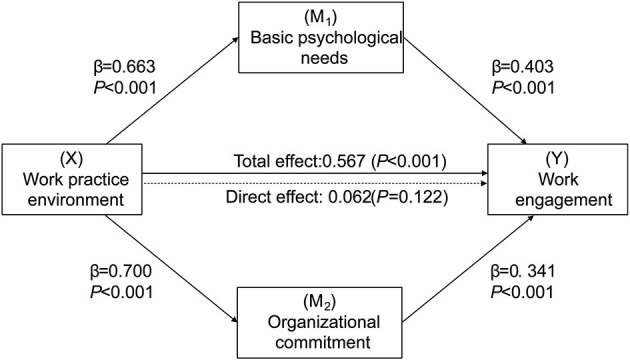
Final model and standardized model paths.

The mediation analysis tests a hypothetical model where one variable X (work practice environment) affects the two mediators, namely, basic psychological needs and organizational commitment, which in turn affect the independent variable Y (work engagement) (Figure 2). The total effect of the work practice environment on work engagement was found to be significant (*B* = 0.567, *SE* = 0.032, *P* < 0.001). The total indirect effect of both mediators (i.e., basic psychological needs and organizational commitment) was statistically significant after controlling for the covariates of demographic characteristics [*B* = 0.505, *SE* = 0.032, 95% *CI* (0.442, 0.566)], while the direct effects of the practice environment on work engagement were not significant (*B* = 0.062, *SE* = 0.113, *P* = 0.122), thus suggesting a significant mediation effect. In other words, the practice environment could affect work engagement indirectly through basic psychological needs and organizational commitment. Moreover, basic psychological needs and organizational commitment jointly accounted for 47% of the variance in nurses' work engagement. Table 3 summarizes the results of the multiple mediation effects.

**Table 3:** Total, direct, and indirect effect estimates.

**Variables**	***B***	***SE***	***P***	**95% Confidence interval**
Total effect of X on Y	0.567	0.032	< 0.001	0.442, 0.567
Direct effect of X on Y	0.062	0.113	0.122	−0.046, 0.393
Total indirect effect X on Y	0.505	0.032	-	0.442, 0.566
Indirect effect *via* M_1_	0.267	0.023	-	0.224, 0.314
Indirect effect *via* M_2_	0.238	0.025	-	0.188, 0.285

95% confidence intervals based on 5,000 bootstrapping samples. X, work practice environment; M_1_, basic psychological needs; M_2_, organizational commitment; Y, work engagement.

## Discussion

This study explored how the work practice environment affects nurses' work engagement. As expected, the work practice environment was positively related to work engagement, and this association was mediated by basic psychological needs satisfaction and organizational commitment. To the best of our knowledge, this is the first study to examine the relationships among these four key variables in one model and to gain a deeper understanding of why the work practice environment influences nurses' work engagement.

One interesting finding was that nurses' work engagement was at the median level and even higher than before the COVID-19 pandemic (37). This is consistent with previous studies (38–40), which demonstrated an increased level of work engagement during the COVID-19 pandemic compared with that before the outbreak of COVID-19. Although the virus poses a challenge in nurses' work practice environment (e.g., a heavy workload, increasing demand for quality care), it does bring an enormous individual and collective effort (39), especially for nurses working in collectivistic cultures, such as China. For example, the government has provided powerful support for health professionals, including nurses, such as free online or offline psychological consultation services, increased compensation, and logistics support (free bus around the hospital) (40). Moreover, nurses try to offer their best face instead of a negative attitude toward work (39) because they are members of the collective; thus, they become more concentrated and active in their work (38). In addition, nurses' strong sense of responsibility and their duties to care for patients made them ignore the difficulties of the extreme circumstances of the COVID-19 pandemic and show a high level of work engagement (41). Surprisingly, the response rate was high in our study, although the data were collected during the COVID-19 pandemic. This may be because our survey was anonymous, which prevented nurses' misgivings from being involved in this study. Moreover, encouragement from nursing administrators and time being allocated for nurses to complete the survey may be another reason for the high response rate.

Our results confirmed a positive relationship between the work practice environment and work engagement, confirming Hypothesis 1; in other words, a healthy nursing practice environment (e.g., supportive nurse leaders, positive nurse–physician relationships, participation in the hospital administration, and adequate nurse staff) was associated with high engagement, which was in line with previous studies (4, 9, 10). A survey conducted by Wan et al. (4) also found that a supportive work environment had a positive impact on nurses' work engagement. A favorable work practice environment can act as a job resource and motivate individuals to dedicate their efforts and energies to their job tasks, which in turn enhance positive psychological states (10), such as work engagement in our study.

An important finding of this study was that the association between the work practice environment and work engagement was mediated by basic psychological needs satisfaction and organizational commitment. The mediation effects indicated that basic psychological needs and organizational commitment play crucial roles in nurses transferring the benefits of the work practice environment to their engagement. These indirect effects revealed the specific psychological mechanism underlying the relationship between the work practice environment and work engagement, helping to broaden our understanding of work engagement and further enhance it.

With regard to the mediating role of basic psychological needs, our results found that basic psychological needs satisfaction mediated the relationship between the work practice environment and work engagement, supporting Hypothesis 2. These results imply that when organizations provide a positive work environment, individuals' basic psychological needs are fulfilled, which subsequently enhances their work engagement. Individuals were more likely to experience a high level of interest in their tasks when they were in a situation that supports basic psychological needs satisfaction (15). As such, a healthy work environment, for instance, harmonious interpersonal relationships and opportunities for ability improvement, could facilitate the fulfillment of psychological needs for autonomy, competence, and relatedness, and nurses are more likely to be motivated and engaged in their work (42). For example, engaging leadership (14), a warm and harmonious humanistic environment (43) and excellent interpersonal relationships (44) were identified as facilitators of satisfying basic psychological needs. When nurses' psychological needs were fulfilled, they tended to show a high level of engagement in their task since basic psychological needs were considered one of the most important motivational factors to enhance work engagement (44). Similarly, Slemp et al. (45) reported that the impact of leader autonomy support (as one kind of work practice environment) on work engagement was mediated by basic psychological needs.

Our study further discovered that organizational commitment also mediated the relationship between the work practice environment and work engagement, confirming Hypothesis 3. Similar results were found by Aharon et al. (46), who identified that the nursing work environment contributed significantly to the explanation of organizational commitment. Previous studies have indicated that those who perceive greater support from their organization (as one component of the work practice environment) are more likely to feel that their organizations care about them, and then a favorable employee-organizational relationship and mutual trust are created (47). Once a favorable employee-organizational relationship was created, nurses were willing to “repay” the organization by devoting their efforts, energy, and time to the work, thus improving work engagement (22). A review conducted by Kim et al. (23) found that eight studies reported commitment as an antecedent to engagement. When individuals are attached and loyal to their organization, they can be immersed in their task and show high work engagement (48).

### Limitations

The data were only collected from Chinese nurses; thus, the results should be cautiously generalized to other nurse groups in different countries or contexts. Due to the cross-sectional survey design, no causal relationships could be established. Social desirability and response biases could not be unavoidable due to the multiple self-report questionnaires. In addition, since the survey was carried out through WeChat, several limitations of web-based surveys may exist, such as respondents not answering truthfully and being faced with technical problems and required abilities (49). We have made many efforts to ensure the authenticity and validity of the data, including setting the duration for answering the questionnaire and only answering the questionnaire once for each participant. Finally, although resampling-based procedures were used, valid statistical inference may be threatened because several study hypotheses were tested (50).

### Implications

The findings of this study highlight the importance of a healthy environment in increasing the level of engagement with work. Therefore, it is recommended that administrators should make efforts to create a favorable work practice environment for nurses, such as increasing nurse participation in hospital affairs, providing adequate resources, and building a mutual trust employee-organizational relationship. However, it is not enough to rely solely on hospital administrators; it requires health systems and hospitals to join together to provide sufficient material and human resources, increase investment in nursing, and eventually build a healthy work practice environment. Moreover, we found that the effects of the work practice environment on work engagement were mediated by basic psychological needs satisfaction and organizational commitment. In other words, the work practice environment must trigger nurses' motivation and then translate it into a high level of engagement. Thus, administrators should satisfy nurses' needs for autonomy, competence, and relatedness to foster their engagement in clinical practice. Autonomy is a threshold issue in nursing practice (4). Empowering leaders were proven to be an important way to fulfill the needs of autonomy (51) since empowering leaders encouraged individuals to discuss problems and control their own work behaviors (52). In addition, providing opportunities for skill improvements, making episodic achievements and recognition more attainable, and creating a harmonious work environment may increase the needs satisfaction of nurses (53). We also suggested that managers could foster nurses' engagement by improving organizational commitment, such as supportive supervisors and colleagues (54), excellent organizational culture (55), and structural empowerment (56). Additionally, nurses could initiatively look for the satisfaction of basic needs by actively learning and searching for collaboration with colleagues (14). With regard to future research, researchers could perform longitudinal studies to verify the effects of the work practice environment, basic psychological needs satisfaction, and organizational commitment on work engagement. In addition, additional studies are needed to replicate this study with other contexts (e.g., primary health care centers), cultures, and countries.

## Conclusions

This study found that the work practice environment was positively associated with work engagement, and this association was mediated by basic psychological needs and organizational commitment. These findings help to explain why the work practice environment influences work engagement in the nursing context and offer newfound knowledge regarding the mechanisms of fostering work engagement among nurses.

## Data availability statement

The raw data supporting the conclusions of this article will be made available by the authors, without undue reservation.

## Ethics statement

The studies involving human participants were reviewed and approved by the IRB of West China Hospital of Sichuan University. The patients/participants provided their written informed consent to participate in this study.

## Author contributions

Y-xN, YW, and G-yY conceived and designed the study and drafted the manuscript. Y-xN, YW, G-yY, YX, and LH contributed to the data collection, statistical analysis, and interpretation of the results. YX and LH revised the manuscript. All authors read and made final approval of the version to be submitted.
